# PuLSE: Quality control and quantification of peptide sequences explored by phage display libraries

**DOI:** 10.1371/journal.pone.0193332

**Published:** 2018-02-23

**Authors:** Steven Shave, Stefan Mann, Joanna Koszela, Alastair Kerr, Manfred Auer

**Affiliations:** School of Biological Sciences and Edinburgh Medical School: Biomedical Sciences, University of Edinburgh, The King’s Buildings, Edinburgh, Scotland, United Kingdom; AC Camargo Cancer Hospital, BRAZIL

## Abstract

The design of highly diverse phage display libraries is based on assumption that DNA bases are incorporated at similar rates within the randomized sequence. As library complexity increases and expected copy numbers of unique sequences decrease, the exploration of library space becomes sparser and the presence of truly random sequences becomes critical. We present the program PuLSE (Phage Library Sequence Evaluation) as a tool for assessing randomness and therefore diversity of phage display libraries. PuLSE runs on a collection of sequence reads in the fastq file format and generates tables profiling the library in terms of unique DNA sequence counts and positions, translated peptide sequences, and normalized ‘expected’ occurrences from base to residue codon frequencies. The output allows at-a-glance quantitative quality control of a phage library in terms of sequence coverage both at the DNA base and translated protein residue level, which has been missing from toolsets and literature. The open source program PuLSE is available in two formats, a C++ source code package for compilation and integration into existing bioinformatics pipelines and precompiled binaries for ease of use.

## Introduction

The discovery of molecular probes or tools, including inhibitors, activators or simply binders of biologically relevant entities is highly valuable and enables the probing of biological networks. Efforts in this area have been greatly accelerated by the application of phage display technology[1–6], a high throughput technique commonly used to find peptidic interactors. Binders and modulators of proteins, protein-protein, and protein-peptide interactions are identified from screening bacteriophages which express a genetically encoded combinatorial peptide library on their surface. Bacteriophages provide a physical link between the encoding DNA and the expressed ligand. The randomized peptide sequence may be of various lengths, allowing biological and chemical exploration of peptides (cyclic and linear)[7–10], antibodies[11–16], and biosimilars [17–20]. High-complexity phage libraries are screened against target proteins in an affinity selection process called biopanning. Biopanning involves incubating the phage display libraries with immobilized target proteins, followed by extensive stringent washing to remove weak-binding and non-bound phages. The remaining bound phages are typically eluted with glycine-HCL and reamplified by infecting host cells. The whole process is typically repeated three to five times to enrich phages with high binding affinity[21, 22]. After biopanning, DNA sequencing allows determination of primary sequence of the binder which can then be synthesized or expressed for use in basic and translational research.

The complexity of a phage display library containing a randomized peptide sequence of a certain length can be defined as the maximum number of unique sequences possible within that library. In a simple 5-mer peptide library made up of the 20 natural amino acids, the complexity is 3.2 million (20^5^) unique sequences. This complexity rapidly increases with increased randomized positions, requiring 1.024x10^13^ unique sequences to fully cover a 10-mer library. Currently, literature routinely reports on libraries of up to 10^10^ unique sequences[4]. In reality, when biopanning, perfect sequence coverage is hardly ever reached. As library complexity increases and larger randomized stretches are expressed, complete library exploration becomes difficult to achieve due to practical limitations, as scientists would rapidly be dealing with volumes and cell numbers unfeasible for even industrial scale laboratories. However, it has been observed by Munoz and Deem that higher affinity antibodies are found through exploration of large high complexity libraries with lower sequence coverage, rather than thorough exploration of low complexity libraries with high sequence coverage[23]. This suggests efforts should be poured into increasing library sequence length (up to a reasonable limit) rather than ensuring that statistically all sequences should be present in a screen, paying the price of reduced sequence length. A critical aspect of sparse exploration of high complexity libraries is that the expressed sequences must be truly random and not skewed to one area of library space. A typical source of skew of a phage display library is enrichment or under-representation of DNA bases at certain positions in the sequence. In a truly randomized library there should be no enrichment for bases globally or at specific positions[24]. This is an important aspect which is not routinely checked in phage display workflows. Next-generation sequencing (NGS) is a powerful tool which can be used to read sequences contained within a sample of a phage display library, typically reporting in the order of 10^6^ sequences. It is critical to note that this random sample containing a fraction of the phage display library is assumed to be representative of the entire library[25]. This sample of the library population does however contain enough information to profile DNA base and peptide residue propensities at each position which could be used to ensure the absence of skew in DNA base distribution.

During our work developing new aspects of phage display technologies, we found no available software which could check and report on potential sources of skew present in NGS reads of phage display libraries. Whilst a multitude of software (commercial and open source) is available to assess the read accuracy of NGS runs, quantifying the accuracy of sequences recovered, no program was available for the task of ensuring that randomized stretches of phage display libraries were truly random. We identified an approach taken by Rodi et al. in which sequencing and inspection of 100 clones in a non-automated manner was used to assess diversity present in phage display libraries[26]. However, Makowski and Soares argue that a more accurate measure of diversity can be achieved by automated analysis of large pools of sequencing data accessible with NGS techniques[27]. Although NGS aware tools such as fastqc (http://www.bioinformatics.babraham.ac.uk/projects/fastqc) do report per base sequence content and PHASTpep counts occurrences of unique sequences between biopanning rounds[28], currently to our knowledge, no tool exists to put these sequences in the context of codon and amino acid frequencies as suggested by Makowski, with or without a dynamic reading frame necessary for the quality control of phage display libraries.

Here we present our new software tool; PuLSE (Phage Library Sequence Evaluation), capable of filling this need by exploiting information present in a sample from a phage display library to build up DNA base propensities at each randomized position. PuLSE is freely available from https://github.com/stevenshave/PuLSE as a free open source package under the MIT license. Using PuLSE, library sequencing output in the fastq file format can be analyzed to determine the positional and overall distribution of DNA bases and resultant amino acid propensities, calculating enrichment factors over the expected ideal. The reading frame is dynamically identified by use of up and downstream markers for forward and reverse sequence reads, performing a dynamic alignment for each sequence read to identify randomized library positions. Uniquely, PuLSE is reading frame aware, able to dynamically locate the start of randomized library stretches, a feature essentially required for the analysis of phage display libraries and not present in other sequence analysis software. In addition, PuLSE may be easily adapted to phage display systems which employ nonsense suppression[29], and allows assignment of any DNA base triplet to custom amino acid residue encoding.

## Materials and methods

### Overview

Initial prototyping of PuLSE was carried out using both the R programming language and Python, which are both commonly used languages for bioinformatic sequence analysis. However, runtimes for data analysis with these interpreted languages quickly became prohibitive as NGS dataset sizes were increased. Experimentation revealed the need for the speed and efficiency of a fast compiled language to process typical phage library NGS data on modest desktop hardware. PuLSE was rewritten in C++14 making use of the standard template library and tested with GCC 5.4.0, Clang 3.8.0 and Visual C++ 19. The authors recognize that distribution of C++ source code could act as a barrier for use by non-specialists, however a standardized build system has been used which automates the compilation process on Linux-based systems and Visual Studio 2015 project files are available for compilation on Microsoft Windows® systems using Visual C++. To further mitigate this problem, precompiled binaries for a variety of platforms are also made available. PuLSE outputs a HTML formatted report at the end of each analysis run which may be opened with any modern HTML5 compliant web browser. In addition, PuLSE also outputs an easily parsable comma separated text file for extraction of results and inclusion into existing analysis pipelines or aggregation packages such as MultiQC[30]. Runtime, including report generation is in the order of 15 seconds for 2.5 million sequence reads of a randomized 5-mer library within a 2.1 GB fastq file using moderate 2016 desktop PC hardware.

The PuLSE implementation is accompanied by an exemplar dataset containing 2.5 million sequence reads from a 5-mer cyclic peptide library and details of how to run the example and interpret the output data. In addition, PuLSE has been extensively tested with simulated NGS data for linear and monobody libraries. The real world NGS data accompanying the PuLSE distribution representing a cyclic 5-mer peptide library. This library was constructed using a modified version of the M13 bacteriophage pSEX81 phagemid [31], following the method described in NEB’s Ph.D.™ Phage Display Libraries manual. The host bacterial strain used for the phage production was *E*.*coli* ER2738. Oligonucleotides (purchased from Sigma Genosys) used to create the library were the forward extension oligonucleotide 5’TGCTGGCAGCTCAGCCGGCCATGG 3’ and the reverse library oligonucleotide 5’ AAAGTTACTGCAGCACANNNNNNNNNNNNNNNGCAAGCCGCCATGGCCGGCTGAGCTGCC 3’. 260 pmol of library oligonucleotides were annealed with a 3 molar excess of the extension oligonucleotide. After annealing, the duplex was extended with a Klenow fragment (NEB #M0210). After extension and deactivation of the Klenow fragment the duplex was restriction digested with NcoI and PstI restriction enzymes (FastDigest^®^, Thermo Scientific). Following the restriction digest the duplex was purified over a 10% non-denaturing PAGE gel. The digested and purified duplex was extracted from the gel using the “crush and soak” method with 300 mM sodium acetate, pH 8, 1 mM EDTA by phenol/chloroform extraction and ethanol precipitation. 3.93 pmol (15 μg) of vector (5876 bp), restriction digested with NcoI and PstI, were, ligated with the duplex in a vector insert ratio of 1:5 and 3 Weiss units / 100 ng vector of T4 DNA Ligase (Thermo Scientific) and incubated overnight at 16°C. The ligation product was purified by phenol/chloroform extraction and ethanol purification. The ligation product (2.62 pmol, 10 μg) was transformed into electrocompetent *E*.*coli* ER2738 carrying the helper phage plasmid M13KO7ΔpIII (partial deletion of the *pIII* gene, Kanamycin resistant) at 30 x 3μl ligation product per 300 μl cell suspension in 2 mm cuvettes using a Biorad Micropulser, program EC2. The electroporations were pooled in a total volume of 100 mL 2xTY medium (50 μg/ml Kanamycin) and incubated with 180 rpm agitation at 37°C for 30 minutes. After this, a dilution series was plated out onto LB agar plates (100 μg/ml Ampicillin) and incubated over night at. 37°C. The pooled electroporations were divided into 4 flasks with 1 L of 2xTY medium (50 μg/ml Kanamycin) and incubated with 180 rpm agitation at 37°C for 2 hours. Ampicillin was then added to achieve a final concentration of 100 μg/ml and incubated with 180 rpm agitation at 30°C over night. The dilution series showed 1.87x10^9^ bacterial hosts had taken up phagemid library constructs (colony forming units). Phages were harvested with isoelectric point precipitation[32]. The cultures were centrifuged and the pH of the clear supernatant was adjusted to pH 4.2. DNA of ~10^12^ phages from the display library was purified by phenol/chloroform extraction and ethanol precipitation. PCR with forward 5’ ATTCATTAAAGAGGAGAAATTAACCATG 3’ and reverse 5’ CGTCATCGTCTAACTTTAAATAATTGG 3’oligonucleotides using Phusion DNA polymerase (NEB, #M0530S) was applied to produce a 188 bp amplicon, carrying the randomized 5-mer sequences. The amplicon was purified via non-denaturing PAGE, extracted from the gel by “crush and soak” followed by phenol/chloroform extraction and ethanol precipitation. A total of 41 pmol (5 μg) amplicon DNA was sent to Otogenetics (Atlanta, USA) for Illumina HiSeq NGS.

### Algorithm description

Design and testing of PuLSE has been undertaken using both real and simulated NGS datasets. These simulated datasets representing linear 7 and 12-mer libraries along with a monobody library were generated using the PuLSE-SimilateDataset program which accompanies the main PuLSE software package. These simulated datasets contain perturbed sequences and edge cases designed to test stability and error handling of NGS software. In addition, the dataset which accompanies the PuLSE program distribution is a real-world NGS dataset representing a cyclic 5-mer peptide library. This library is designed to express peptides consisting of two cysteine residues flanking a stretch of 5 random amino acids which are then cyclized through a disulfide bridge, and contains 2.5 million sequence reads. This real world NGS dataset will be used throughout this algorithm description. The PuLSE program is a command line utility with no graphical user interface, requiring minimally only two inputs. These are the fastq data filename containing library reads and a forward strand library definition (FSLD). NGS read data is often supplied in a compressed format, using Gzip compression. PuLSE is able to read Gzip compressed fastq files, making extraction of large datasets unnecessary, reading directly from the compressed data file.

### Forward strand library definition (FSLD)

**Fig 1:**
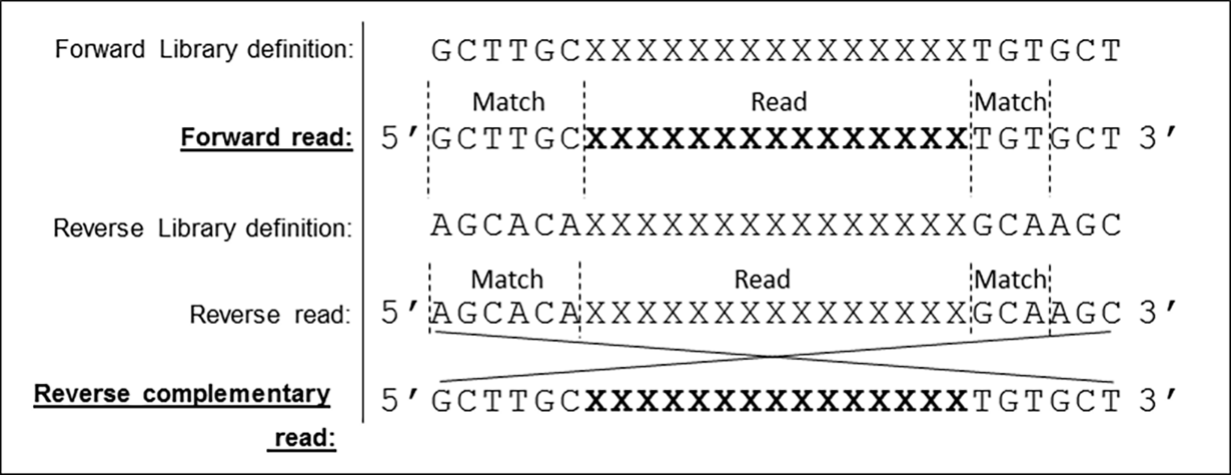
Library definition format. Example of the library definition format allowing robust identification of randomized positions within a sequence from forwards and reverse complementary strand reads.

The FSLD is used to dynamically identify the randomized portion of a library within NGS sequence read data, acting as a mask which is overlaid throughout each read sequence and queried as to the presence of known start and end flanking sequences. If present, then the DNA bases in between these start and end markers may be taken as the randomized portion of the library for further analysis. PuLSE supports all IUPAC nucleotide codes (A, C, G, T, R, Y, S, W, K, M, B, D, H, V, N), allowing the definition of restricted codon sets. See the PuLSE software package for a full table of allowable codes. In addition to the standard nomenclature for any base, for legacy reasons, PuLSE allows the use of ‘X’ to represent any nucleotide base, the equivalent of the IUPAC nucleotide code ‘N’. The FSLD for the exemplar dataset may be defined as follows: “CGTTGCXXXXXXXXXXXXXXXTGTGCT”. In this library, the initial CGTTGC motif acts as the upstream marker for the beginning of a randomized stretch of 15 bases denoted by ‘X’ and encoding 5 amino acids. Initial versions of the algorithm matched the start marker and read the specified number of following randomized bases, however, we noticed a large proportion of stop codons present in the final analysis of non-quality controlled read data, signifying that either sequence miss-reads or frame shifts after upstream marker identification took place which should not, and cannot be included in positional occurrence statistics. We therefore introduced a downstream marker. In the example above, the first 3 bases of the downstream marker ‘TGT’ must be present after the randomized sequence to be considered a good library read. Note that only the first three bases of the downstream marker must be present in a sequence read for the randomized portion to be accepted. It is worth noting however, that even though three bases of the downstream marker are ever used, it must be specified with the same length as the upstream marker. This is to enable the reading of reversed sequence reads, where the downstream marker is converted into an upstream marker and the upstream into a downstream marker. This is essential as reverse DNA strand reads may also be detected by PuLSE. Up and down-stream markers before and after the randomized sequences may be of any length, however, only the first three end marker bases are ever used (See Fig 1).

### Codon remapping

Additionally, more arguments may be passed to PuLSE to remap the standard DNA triplet to protein residue mapping. This is useful when working with phage display systems employing nonsense suppression. The standard DNA codons used by PuLSE to translate to amino acid residues are as follows: “TTT:F, TTC:F, TTA:L, TTG:L, CTT:L, CTC:L, CTA:L, CTG:L, ATT:I, ATC:I, ATA:I, ATG:M, GTT:V, GTC:V, GTA:V, GTG:V, TCT:S, TCC:S, TCA:S, TCG:S, AGT:S, AGC:S, CCT:P, CCC:P, CCA:P, CCG:P, ACT:T, ACC:T, ACA:T, ACG:T, GCT:A, GCC:A, GCA:A, GCG:A, TAT:Y, TAC:Y, TAA:*, TAG:*, TGA:*, CAT:H, CAC:H, CAA:Q, CAG:Q, GAA:E, GAG:E, AAT:N, AAC:N, AAA:K, AAG:K, GAT:D, GAC:D, TGT:C, TGC:C, TGG:W, CGT:R, CGC:R, CGA:R, CGG:R, AGA:R, AGG:R, GGT:G, GGC:G, GGA:G, GGG:G”. Translational readthrough or amber stop codon suppression is a feature whereby instead of the DNA triplet UAG mapping to the amber stop codon, it incorporates a glutamine residue to be expressed in the system. This codon change can be passed to PuLSE by simple inclusion of “UAG Q” after all other arguments. There is no limit to the number of changes which can be made to PuLSE’s internal codon table, allowing complete customization through specification of multiple instances of a DNA triplet followed by an amino acid residue letter. For further usage instructions, please see instructions within the PuLSE distribution package.

### Outputs

PuLSE output comes in the form of a formatted HTML report, along with an easily parsable tab separated text file for inclusion of PuLSE results in automated pipelines. Both the HTML formatted and plaintext outputs contain six distinct sections.

Run information: An overview of parameters describing the PuLSE quality control run, including the input fastq name, the library definition string, derived forward, backwards upstream and downstream markers, the number of randomized DNA base positions expected and a list of any non-standard DNA codon mappings used, and any custom triplet mappings employed in the analysis.

Basic statistics: Counts of successful reads from the input fastq file, the number of unique DNA and protein sequences found, along with the number of unique protein and DNA sequences not found. NGS sequence reads normally do not cover all sequences within a phage display library and represent only a sample of the entire population. For this reason, the number of sequences not found in a large library will, always be high.

Cumulative counts: This output section is included as a legacy technique for evaluating phage display library diversity, and is superseded by newer PuLSE evaluation methods. This evaluation method counts the number of times a unique sequence (both DNA and peptide) is found. This analysis method is only valid for smaller libraries, in which the sample taken for NGS sequencing can be expected to cover the whole library multiple times. In a small library, where the number of reads greatly outweighs the number of unique peptides present, we would expect to see a normally distributed population centered around the number of reads divided by the theoretical library complexity. Where read size is a fraction of the number of possible unique sequences, we typically observe that most sequences are missing and a large proportion of sequence reads are found only once as expected. The cumulative count allows quick evaluation of high coverage, low complexity libraries.

Most common sequences: In rank order, the 100 most commonly observed DNA and protein sequences are given along with their associated counts. This section enables a quick check to see if any sequences are highly expressed by the phage display system. If outliers are found and a sequence is repeated multiple times, then the library may be compromised with biased expression or non-randomized base occurrences.

Protein residue counts: Counts of protein residue occurrences by position within the library. Ideally, occurrences of amino acids in each position would be proportional to the number of codons mapping to unique protein residues. For example, in the standard DNA triplet to protein residue map, arginine may be made from six triplets (CGT, CGC, CGA, CGG, AGA and AGG), whereas methionine by only one (ATG).

DNA base counts: Counts of occurrences for the four DNA bases within each randomized position within the library. Occurrence counts in a random library should be close to the number of valid NGS sequence reads found by PuLSE divided by four.

Normalized peptide residue heatmap: This output can be considered the most valuable analysis method undertaken by PuLSE. Here, the normalized occurrences of peptide residues by position within the library are reported. A value of 1.0 indicates that a peptide residue was observed at exactly the expected rate at a certain library position. A score greater than one indicates enrichment of protein residue occurrence. Similarly, a score less than one indicates a residue was present less than expected. Critically, the expected occurrence rates for each peptide residue used in this output are calculated from the DNA codon to peptide residue translation table used in the PuLSE run; if the user has customized the table with additional command line arguments or used a restricted codon set, then those changes are captured here and the correct, non-standard rates used. This is essential to report the correct rates of systems employing nonsense suppression. In the HTML formatted output of PuLSE, this analysis is color coded in heatmap format, indicating enrichment (red), expected (white) and underrepresentation (blue) of occurrences.

Normalized DNA base heatmap: Similar in purpose to the normalized protein residue heatmap and present in heatmap format in PuLSE’s HTML formatted output, enrichment of DNA base occurrences at each position within the library is reported. The expected inclusion rate is simply the number of valid NGS reads divided by their expected occurrence, derived from the FSLD.

## Results and exemplaric use

**Fig 2:**
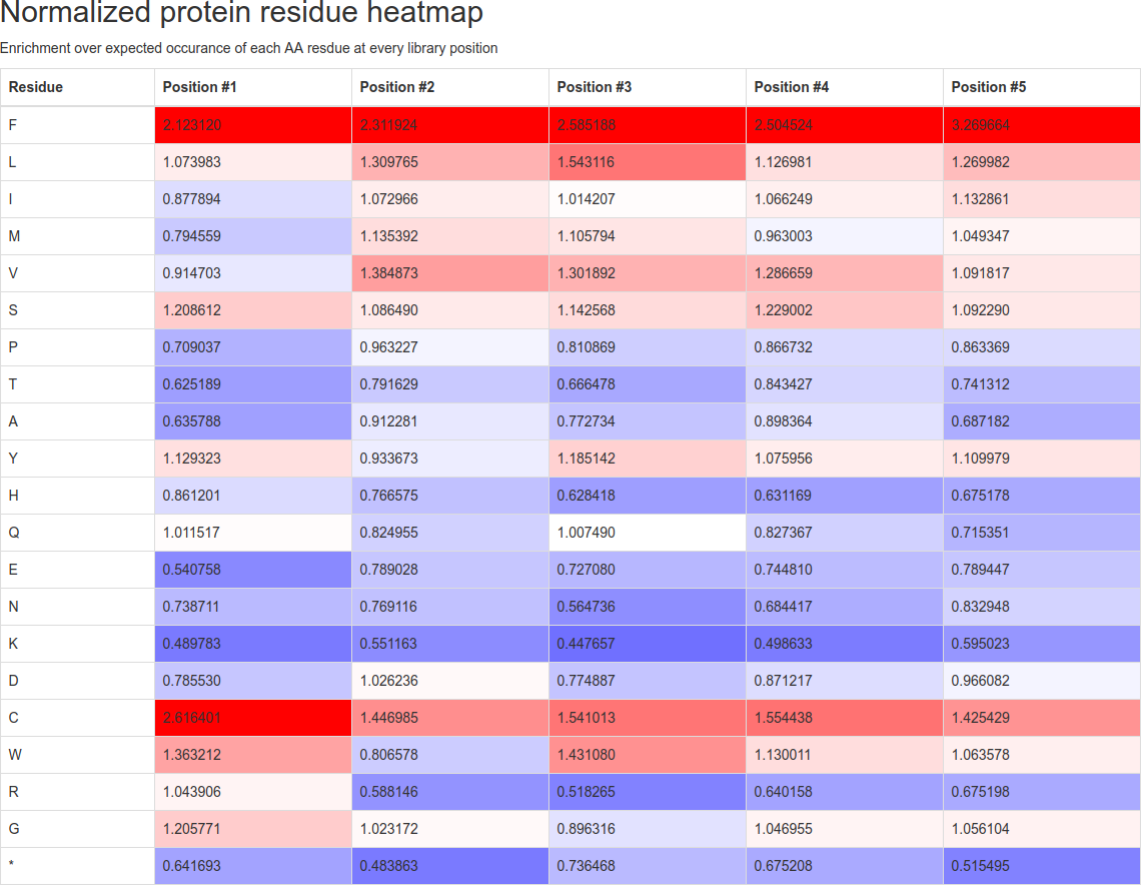
Example protein residue occurrence heatmap. Protein residue occurrence heatmap for the exemplar dataset accompanying the PuLSE software distribution. Phenylalanine is slightly enriched over its expected occurrence rate for each position within the library. Lysine is underrepresented at each position. However, the enrichment and underrepresentations are not pronounced, ranging from 0.44 to 3.27 of expected.

**Fig 3:**
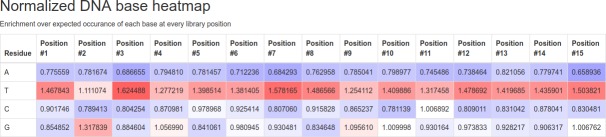
Example DNA base occurrence heatmap. DNA base occurrence heatmap for exemplaric dataset accompanying the PuLSE software distribution. Enrichment and underrepresentation is not pronounced, suggesting the profiled phage library possesses a high degree of randomness and therefore the expected diversity.

NGS sequencing data for a cyclic 5-mer peptide library; “sample-pulse-5merCyclic-CGTTGCXXXXXXXXXXXXXXXTGTGCT.fastq.gz” accompanies the PuLSE program distribution. Due to distribution size limitations, this truncated NGS dataset was generated by taking the first 10 million lines from a NGS run. This dataset represents exactly 2.5 million sequence reads in Sanger / Illumina 1.9 encoding scheme. As previously described, the FSLD for this library is “CGTTGCXXXXXXXXXXXXXXXTGTGCT”, specifying 15 randomized DNA base positions which will be translated to 5 randomized amino acids flanked by cysteines (TGC and TGT). Our phage expression system implements amber stop codon suppression, whereby the usual UAG stop codon expresses a glutamine residue. The command line for a PuLSE run on this data would therefore take the following form: “pulse sample-pulse-5merCyclic-CGTTGCXXXXXXXXXXXXXXXTGTGCT.fastq.gz CGTTGCXXXXXXXXXXXXXXXTGTGCT UAG Q”. PuLSE’s runtime on this system containing 2.5 million reads and output generation is of the order of 8 seconds on modest 2016 hardware. Interestingly, performance is worse on non-compressed data, taking of the order of 15 seconds to operate on the decompressed fastq file. This is due to the high decompression speed of Gzip compression reducing the amount of slow disk access required. Running the above command produces two output files: “sample-pulse-5merCyclic-CGTTGCXXXXXXXXXXXXXXXTGTGCT.html” and “sample-pulse-5merCyclic-CGTTGCXXXXXXXXXXXXXXXTGTGCT.txt”. The generated HTML report may be viewed in any modern web browser supporting the bootstrap 3 component library. From analysis of the exemplar dataset, PuLSE finds 1,070,319 valid sequences. Valid sequences are defined as containing 15 randomized positions between upstream and downstream markers. Sequences are searched in both forward and reverse directions. 972,372 unique DNA sequences are found, representing just 0.09% of the entire theoretical library. These DNA sequences translate to 644,214 unique protein sequences which covers 20.13% of possible protein sequences encodable in the library. As expected, the cumulate counts section shows that most DNA and peptide sequences are not found. Interestingly, however, one peptide sequence is found 105 times. The identity of this sequence is revealed in the most common sequences table and is shown to be LLLSS. Finally, the last two heatmap colored tables shown in Fig 2 and Fig 3 give an overview of how unbiased and truly random the sequenced library is.

Phenylalanine is shown as the most enriched residue, with enrichments of 2.12, 2.31, 2.59, 2.50 and 3.27 of expected for each of the five positions. The most under represented residue is lysine, represented 0.49, 0.55, 0.45, 0.50 and 0.60 of expected over each position.

The normalized DNA base occurrence heatmap shows little variation in residue occurrences, varying between a minimum of 0.66 for adenine in position 15 to a maximum of 1.62 for thymine in position three. Due to the narrow range of occurrence factors for both the protein residue and DNA bases, we may conclude that this is a high-quality library with little skew present. If evaluation had revealed residues present at a fraction of expected, or a high number of multiples more than expected, then we could call the library randomness into question and proceed with a repeat of NGS after thorough sample mixing to confirm the skew present. Data within these tables may be used by users for further statistical analysis.

## Discussion

We identified the need for a software package which gives a thorough overview of phage library quality measured by the observed distribution of DNA bases and triplets corresponding to amino acids. PuLSE has been thoroughly tested on simulated and real world experimental phage display screening library data and found to fill a previously unmet need. It is truly surprising that no available software package could perform the evaluation required for QC of phage library randomness by NGS data. Development of PuLSE will continue under the existing permissive open source license. It is hoped that wider adoption of PuLSE into regular use will enable future advancements in analysis techniques and integration into standard pipelines.
